# Effects of Global and Specific DNA-Binding Proteins on Transcriptional Regulation of the *E. coli bgl* Operon

**DOI:** 10.3390/ijms231810343

**Published:** 2022-09-07

**Authors:** Dennis Tran, Zhongge Zhang, Katie Jing Kay Lam, Milton H. Saier

**Affiliations:** School of Biological Sciences, Department of Molecular Biology, University of California-San Diego, La Jolla, CA 92093, USA

**Keywords:** *bgl* operon, β-glucosides, H-NS, StpA, Crp, Fis, DNA loop, insertion sequences (IS)

## Abstract

Using reporter gene (*lacZ*) transcriptional fusions*,* we examined the transcriptional dependencies of the *bgl* promoter (P*bgl*) and the entire operon regulatory region (P*bgl*-*bglG*) on eight transcription factors as well as the inducer, salicin, and an IS5 insertion upstream of P*bgl*. Crp-cAMP is the primary activator of both P*bgl* and the *bgl* operon, while H-NS is a strong dominant operon repressor but only a weak repressor of P*bgl*. H-NS may exert its repressive effect by looping the DNA at two binding sites. StpA is a relatively weak repressor in the absence of H-NS, while Fis also has a weak repressive effect. Salicin has no effect on P*bgl* activity but causes a 30-fold induction of *bgl* operon expression. Induction depends on the activity of the BglF transporter/kinase. IS5 insertion has only a moderate effect on P*bgl* but causes a much greater activation of the *bgl* operon expression by preventing the full repressive effects of H-NS and StpA. While several other transcription factors (BglJ, RcsB, and LeuO) have been reported to influence *bgl* operon transcription when overexpressed, they had little or no effect when present at wild type levels. These results indicate the important transcriptional regulatory mechanisms operative on the *bgl* operon in *E. coli*.

## 1. Introduction

The *E. coli bgl* operon encodes BglG, BglF, and BglB that are involved in operon regulation and the utilization of aromatic β-glucosides, salicin, arbutin, and esculin, as well as non-aromatic β-glucosides such as cellobiose, as carbon sources [1,2]. The first gene *bglG*, formerly named *bglC* [3], flanked by two Rho-independent terminators, encodes an antiterminator protein whose function is to prevent the formation of terminator structures, stabilizing the 5′ end of the *bgl* mRNA and enabling operon transcription [4,5]. The second gene, *bglF*, codes a phosphoenol pyruvate-dependent phosphotransferase system (PTS)-dependent enzyme/transporter essential for β-glucoside uptake and phosphorylation, but it also plays a regulatory role, controlling the antitermination process [3,6,7,8]. The third gene in the operon, *bglB,* codes for a phospho-β-glucosidase that is responsible for hydrolyzing phosphorylated aromatic β-glucosides, such as salicin-P, arbutin-P, and esculin-P, allowing it to release glucose-6-P and the aglycone [2,9].

Although the *bgl* operon is found to be expressed in *E. coli* infecting mouse livers [10], the operon is transcriptionally silent (cryptic) and uninducible by β-glucosides in wild-type *E. coli* strains under standard lab conditions due to the presence of two terminators flanking the *bglG* gene [11] and strong repression by H-NS, the Histone-like Nucleoid-Structuring protein [12,13,14]. A variety of mutations [12,14,15,16,17,18,19,20] can occur in wild type cells after prolonged incubation with β-glucosides, thereby suppressing the silencing state, a process activating transcription. The activated *bgl* operon gives rise to a Bgl^+^ phenotype (which is able to use β-glucosides as the sole carbon source for growth). The most common types of Bgl^+^ mutations are due to insertions of IS (insertion sequence) elements such as IS1 and IS5 upstream of the *bgl* promoter region [2,21], a region carrying a SIDD (Superhelical stress-Induced DNA Duplex Destabilization) structure [22]. IS insertional mutations are found to be enhanced by the presence of the operon substrates salicin or arbutin and positively regulated by BglG [23]. Once the *bgl* operon is activated by a mutation, it becomes inducible. In the absence of β-glucosides, the Crp-dependent promoter initiates transcription, but it subsequently terminates at one of those two terminators flanking *bglG*. In the presence of operon inducers, transcription partially bypasses both terminators and yields mature operon transcripts.

BglG and BglF comprise a sensory system that dictates the termination and antitermination processes of *bgl* operon transcription [24]. The BglF sensor phosphorylates BglG in the absence of β-glucosides, thus inactivating it (operon silencing), while BglF dephosphorylates BglG in the presence of β-glucosides, thus activating it (operon expression). After dephosphorylation by BglF, BglG is phosphorylated by HPr (or FPr) and subsequently forms homodimers that bind to a site in the *bgl* mRNA that partially overlaps the Rho-independent terminators, impeding the formation of terminator hairpin structures and enabling transcriptional readthrough [4,5].

In addition to two intra-operon regulators, BglG and BglF, a number of transcription factors are thought to play roles in regulating *bgl* operon expression. Crp, the cyclic AMP (cAMP) receptor protein, is a global regulator in *E. coli* that binds to cAMP to regulate (usually to activate) the expression of genes involved in carbon utilization [25]. Crp-cAMP regulates more than 180 genes by responding to the changing amounts of intracellular cAMP [26]. As a major activator of the *bgl* operon, the Crp-cAMP complex binds to an upstream site near the promoter to activate it [14,27]. A point substitution within the Crp operator, yielding a more favorable binding site, results in a Bgl^+^ phenotype [15]. BglJ, a LysR-type transcriptional regulator carrying a C-terminal helix-turn-helix motif [17,20], when overexpressed, causes the activation of the H-NS-repressed *bgl* operon [17,28,29]. In wild-type *E. coli* cells, *bglJ* expression is negligible due to the strong repression by H-NS [30]. RcsB, a LuxR-type transcriptional regulator, is a response regulator involved in the regulation of colonic acid capsule synthesis, cell division, motility, and biofilm formation [31,32]. RcsB and BglJ, harboring similar DNA binding domains at their C-termini, can form heterodimers, binding to an upstream promoter region and relieving H-NS-mediated repression of the *bgl* operon [29,33]. LeuO is a global transcription factor that not only regulates the leucine biosynthesis operon of *E. coli* but is also involved in the regulation of stress responses [34,35]. Its overexpression interferes with the silencing of *bgl* by H-NS and thereby activates *bgl* operon transcription, although it is not required for *bgl* activation upon IS insertional mutation or the absence of H-NS [16]. Alternatively, LeuO may indirectly regulate the *bgl* promoter by increasing *bglJ* expression [30]. Similar to *bglJ*, *leuO* expression is subject to repression by H-NS [30].

In addition to those positive regulators described above, the *bgl* operon is subject to the repression of several negative regulators. As the main silencer of the *bgl* operon, the histone-like heat-stable nucleoid structural protein (H-NS) is a major nucleoid protein that is involved in chromosomal stability and transcriptional regulation [36]. H-NS preferentially binds to A/T rich and curved DNA [37,38]. When bound to the DNA, H-NS can self-oligomerize [39] and create a nucleoprotein complex that often represses transcription, either by blocking RNA polymerase binding or by trapping the polymerase [40,41,42]. Therefore, H-NS is often an important negative regulator of transcription, and, as expected, it decreases the transcriptional readthrough of the *bgl* operon. H-NS represses the *bgl* operon by binding to the upstream promoter region and a site within the *bglG* gene [13] in an apparently synergistic fashion [43]. However, thus far, a DNA looping mechanism mediated by H-NS when bound to its two binding sites within the *bgl* operon has not been demonstrated, although the repression of transcriptional initiation by such DNA looping has been reported in several other *E. coli* promoters [40,44,45,46].

H-NS is also involved in regulating physiological adaptation to the environment [47] and it can play a role in regulating transposon-mediated directed mutation, determining how mutations can occur at higher frequencies when beneficial to the organism under stressful environmental conditions ([23]; Lam et al., manuscript in preparation).

StpA, an H-NS paralog, can form heterodimers with H-NS [48,49] and plays a role in gene regulation and silencing [50]. This small nucleoid protein can function as a DNA-binding adaptor that is necessary for repression by a C-terminally truncated H-NS or an H-NS carrying the I119T mutation (both defective in DNA binding) [28,51]. One report suggests that StpA alone does not repress the *bgl* promoter [28].

Fis is a small, abundant nucleoid-associated protein primarily expressed in the exponential phase [52]. Similar to other DNA structural proteins, Fis is capable of binding to and bending the DNA and acts as a global regulator that participates in essential cell processes such as rRNA and tRNA gene transcription [53,54]. In vitro assays showed that Fis is a repressor of the *bgl* promoter, contributing to promoter silencing and antagonizing Crp for promoter activation [55]. Scant research has demonstrated the characterization of the in vivo interaction of these two proteins in *bgl* regulation. Lastly, the stress/stationary phase-response sigma factor RpoS can be involved in *bgl* operon repression [56,57]. RpoS-dependent repression requires the presence of Crl [58], an RNA polymerase holoenzyme assembly factor [59]. In an *rpoS* mutant background, increased levels of BglG conferred a growth advantage to Bgl^+^ cells during the stationary phase [60,61].

In this paper, we examine the transcriptional activities of the *bgl* promoter (P*bgl*) alone (with no terminators) and the entire operon regulatory region (P*bgl*-*bglG*) (the promoter plus the first gene flanked by two terminators) by comprehensively exploring the effects of each proposed regulator introduced above, either singly or in combination. The possible H-NS-mediated DNA looping and the effects of StpA and in vivo Crp/Fis antagonism on *bgl* operon regulation were examined as well. Deletion mutants were constructed for each of these genes, and they were analyzed by measuring their effects on P*bgl* and P*bgl*-*bglG* using a single copy *lacZ* reporter gene located at the *lac* locus (with an intact native *bgl* operon simultaneously present), with and without salicin as the inducer of *bgl* operon expression. Our results show that Crp is essential for both the promoter’s and the operon’s activities. As expected, H-NS is the major silencer of the operon, but it is a weaker repressor of the promoter. H-NS may exert its inhibitory effect by binding to the promoter and the *bglG* gene and looping the DNA. IS insertions dramatically increase *bgl* operon transcription in the presence of β-glucosides, but they only slightly enhance promoter activity. StpA moderately represses the *bgl* operon in the absence of H-NS, while Fis exerts a more recessive effect in the absence of Crp. β-glucosides have no effect on the promoter but significantly induce activated *bgl* operon transcription. At the wild type levels, BglJ, RcsB, the BglJ/RcsB combination, and LeuO have negligible effects, although their overexpression leads to a Bgl^+^ phenotype [16,17,29]. The results provide further insight into the importance and possible mechanism that each gene product plays in regulating the expression of the *bgl* operon.

## 2. Results

### 2.1. Crp Strongly Activates the bgl Promoter While H-NS Weakly Represses It

**Figure 1:**
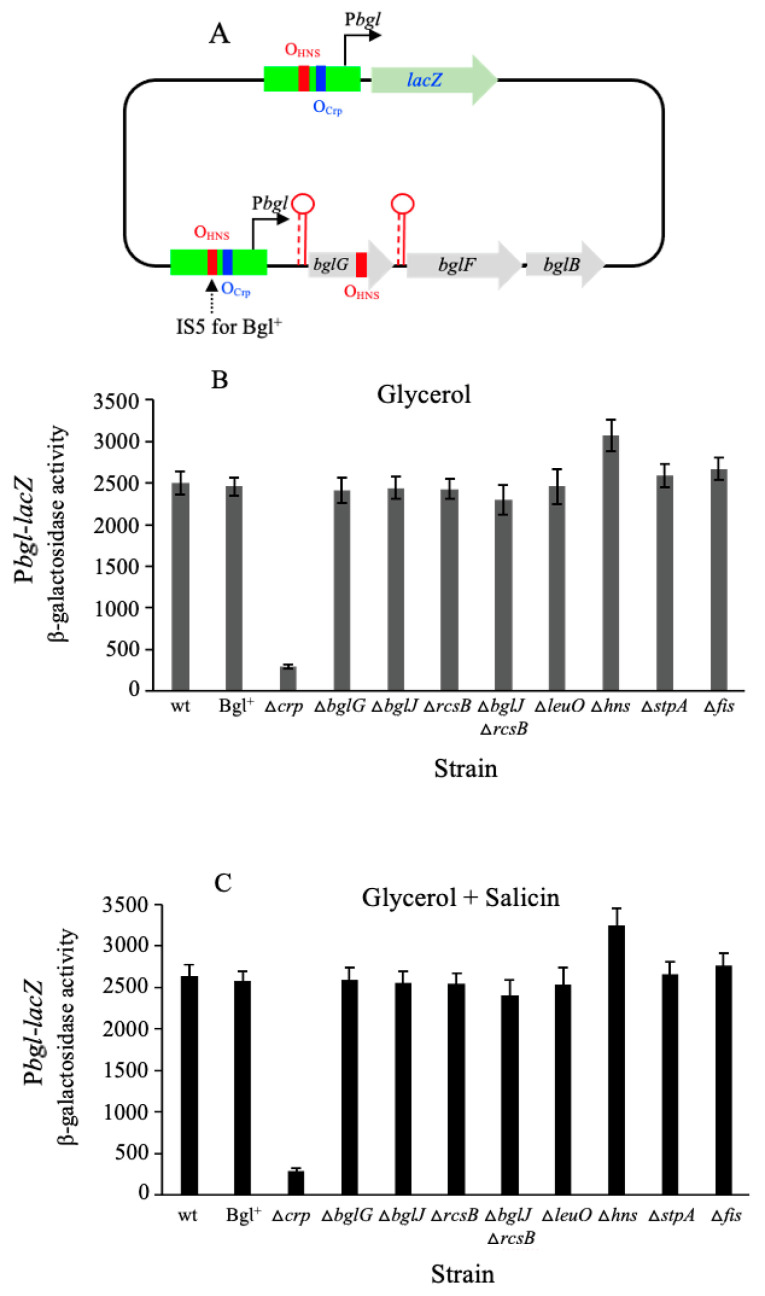
The *bgl* promoter (P*bgl*) activities in the wild type and various isogenic genetic backgrounds. Cells were grown in M63 minimal media with shaking at 37 °C. At least four samples were collected at OD_600_ values of 0.2 to 1.0 during the exponential growth phase. Bacterial samples were subject to β-galactosidase assays as described in Section 4, and the enzyme activities were calculated using the equation [(OD_420_−1.75 × OD_550_)/(sample volume in mL × time in min)] × 1000. For a given test strain, the slope of OD_600_ values versus β-galactosidase activities was referred to as the promoter activity. (**A**) Diagram showing the *lacZ* transcriptional reporter for the *bgl* promoter (P*bgl*-*lacZ*). P*bgl* (−205 to + 54 relative to the transcriptional start site) with no terminators was fused to the upstream region of the *lacZ*’s RBS (that is, to TTTCACACAGGAAACAGCT) at the *lac* locus, replacing *lacI* and the *lacI*/*lacZ* intergenic region. The native *bgl* operon remained intact. However, for strain Bgl^+^, there is an IS5 element oriented in the inverse direction and inserted at −207.5 upstream of the *bglG* translation start site. For both the promoter reporter and the native *bgl* operon, the blue bars represent the Crp binding sites (O_Crp_) while the red bars represent the proposed H-NS binding sites (O_HNS_). (**B**) P*bgl* activities in cells grown with glycerol as the primary carbon source. (**C**) P*bgl* activities in cells grown with glycerol and salicin as carbon sources.

We began our studies on *bgl* operon expression by examining the effects of the genetic deletion of eight transcription factors (previously reported to exert influences on the transcription of this operon) on the *bglGFB* promoter (P*bgl*) activity. Using a P*bgl*-*lacZ* transcriptional reporter integrated within the *E. coli lac* locus while the native *bgl* operon remained intact (Figure 1A), we measured the promoter activities in the presence and absence of each of these eight transcriptional factors. The cells were first cultured with glycerol as the carbon source; only two transcription factors exerted appreciable effects on P*bgl* activity: Crp, an activator, and H-NS, a repressor (Figure 1B). While the dependency on Crp was great (a 10-fold reduction in the absence of Crp; see Figure 1B columns 1 and 3 from the left), the dependency on H-NS was small (only a 20% increase in the absence of H-NS; see Figure 1B, columns 1 and 9). This result presumably reflects the fact that the H-NS binding site in the *bglG* gene is not present as this gene was replaced by the *lacZ* reporter gene (see Section 3). All other transcription factors had negligible effects on the promoter activity in their presence versus their absence. This was also true when glycerol plus salicin served as the carbon sources during bacterial growth (compare Figure 1B with Figure 1C). This result showed that salicin, a potent inducer of *bgl* operon expression, had essentially no effect on *bgl* promoter activity under the conditions used in this study (see the Section 4).

### 2.2. H-NS Is the Strong Dominant Repressor of the bgl Operon

**Figure 2:**
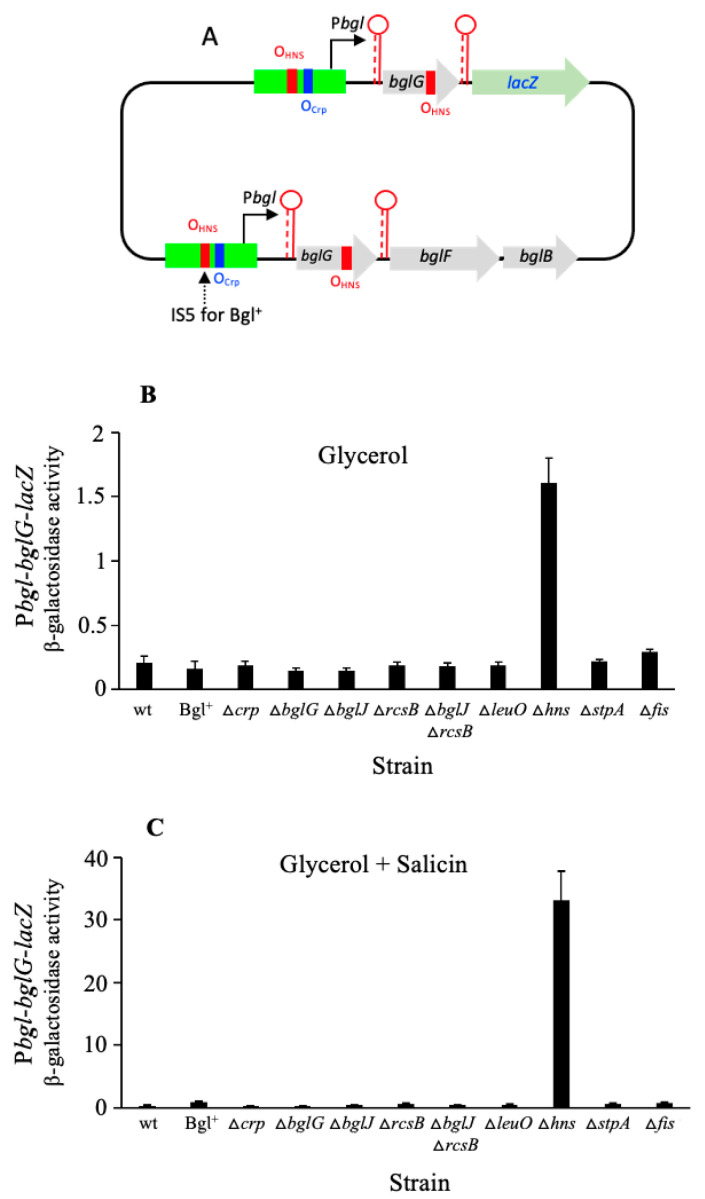
The *bgl* operon activities in the wild type and its various genetic backgrounds. Culture preparation, sample collection, and β-galactosidase assays were carried out as in Figure 1 (see Section 4). (**A**) Diagram showing the *lacZ* transcriptional reporter for the entire regulatory region of the *bgl* operon (P*bgl*-*bglG*-*lacZ*). The region carrying P*bgl* and *bglG*, including both terminators (−205 to + 1127 relative to the *bglG* transcriptional start site), was fused upstream of the *lacZ*’s RBS (that is, TTTCACACAGGAAACAGCT) at the *lac* locus. Strain Bgl^+^ carries an IS5 element oriented in the inverse direction and inserted at −207.5 upstream of the *bglG* translation start site. For all other strains, the native *bgl* operon remains unchanged. For both the operon reporter and the native *bgl* operon, the blue bars represent the Crp binding sites (O_Crp_) while the red bars represent the proposed H-NS binding sites (O_HNS_). (**B**) *bgl* operon activities in cells grown in M63 with glycerol as the primary carbon source. (**C**) *bgl* operon activities in cells grown with glycerol and salicin as carbon sources.

The above experiments were repeated using a *bgl* operon reporter (P*bgl*-*bglG*-*lacZ*) integrated at the *lac* locus, in which the regulatory region of the *bgl* operon, including P*bgl* plus the *bglG* gene, flanked by the two terminators, preceded the fused *lacZ* gene (Figure 2A). The results obtained using this construct are recorded in Figure 2B,C. In the absence of salicin, *bgl* operon expression was minimal due to the presence of both terminators that efficiently block transcriptional readthrough (Figure 2B). However, deletion of the *hns* gene still enhanced the expression of the operon about eightfold (see columns 1 and 9 of Figure 2B), indicating that with no H-NS binding, those two terminators flanking *bglG* are not sufficient for the complete abolition of *bgl* operon expression. In the presence of salicin, the deletion of *hns* dramatically enhanced *bgl* gene expression, up to 100-fold (compare columns 1 and 9 in Figure 2C). These results indicate that H-NS is the major repressor of the *bgl* operon, regardless of the presence of salicin.

All other transcription factors examined had only a small effect or were essentially without an effect (Figure 2B,C). For Bgl^+^ cells, the native *bglGFB* operon should have a significantly increased expression by allowing for the entry of salicin into the cell cytoplasm. However, the operon reporter, P*bgl*-*bglG*-*lacZ*, at the *lac* locus, remained silent in these Bgl^+^ cells grown with salicin since there is no IS insertion present in the reporter construct (Figure 2A and column 2 of Figure 2C). The results obtained clearly suggest that salicin enhances the expression of the operon, although it had no effect on the promoter strength or H-NS repression. Moreover, salicin did not promote regulation by any of the other transcription factors examined.

We also determined whether these transcriptional regulators significantly affect *bgl* operon expression in the absence of H-NS. These activators and repressors were individually deleted in a ∆*hns* background. As usual, all the double mutants were cultured with glycerol and salicin, and their operon activities were measured using the operon reporter, P*bgl*-*bglG*-*lacZ*. As shown in Supplementary Figure S1, the deletion of *crp* almost abolished *bgl* operon expression (column 2), confirming that Crp is the primary positive regulator when the operon is activated by removing H-NS. However, the absence of all other transcription factors still had only small effects (≤10% changes) on operon expression. These results together with those described in Figure 2 indicate that except for Crp and H-NS, all other transcription factors, when expressed at their wild-type levels, play minor roles in regulating the *bgl* operon, both in the presence and in the absence of H-NS.

Our studies were extended by examining the consequences of the increased expression of *bglG* by using additional strong promoters (Supplementary Figure S2), comparing the transcriptional rates when the wild type *bgl* promoter was used, versus the stronger *lacI*q promoter, or the very strong *tet* promoter. As it can be seen, the overexpression of *bglG* only increased the operon expression two to threefold, in either the absence (left panel) or the presence (right panel) of salicin. This is consistent with our previous observation that such low levels of residual operon expression due to *bglG* overexpression remains Bgl^−^ [23].

The above-described measurements were conducted using cells from the exponential growth phase. We next examined *bgl* operon expression during the stationary phase with or without RpoS, the stationary phase sigma factor. Supplementary Figure S3 (left panel) shows that using the wild-type cells, *bgl* operon expression remains silent from the middle to the late stationary growth phase. This is also true for the cells deleted for *rpoS* (right panel of Supplementary Figure S3), although it recognizes RpoD (sigma 70) promoters [62,63]. The loss of RpoS had a negligible impact on the *bgl* operon-silencing state (when comparing the left panel and the right panel). This is not consistent with the previously reported literature [56,57].

### 2.3. StpA Represses the bgl Operon Only in the Absence of H-NS

**Figure 3:**
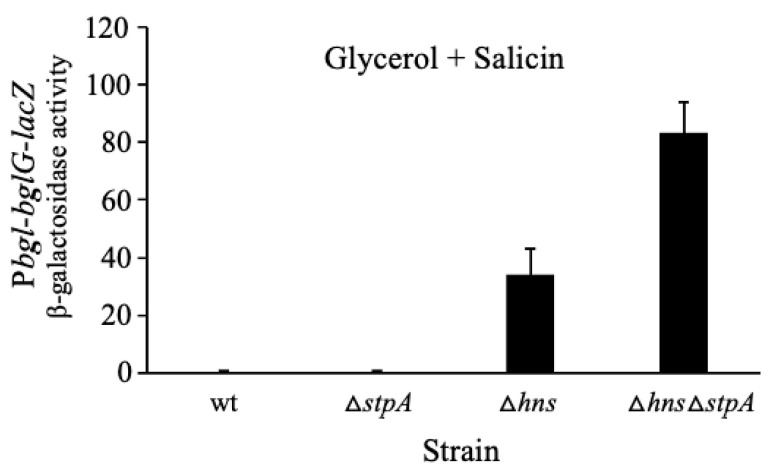
Inhibitory effect of StpA on *bgl* operon expression in the absence of H-NS. Using the operon reporter, P*bgl*-*bglG*-*lacZ* (at the *lac* locus), the *bgl* operon transcriptional activities were assayed, comparing the *stpA* single mutant (∆*stpA*), the *hns* single mutant (∆*hns*), and the *hns*/*stpA* double mutant (∆*hns*∆*stpA*). Cells were cultured in M63 with glycerol and salicin. Sample preparation and β-galactosidase assays were carried out as in Figure 1.

We have shown that the deletion of the *stpA* gene did not exert an effect *on bgl* operon expression when the *hns* gene was intact (Figure 2; also see the first two columns of Figure 3). However, in the absence of H-NS, the deletion of the *stpA* gene (that is, strain ∆*hns*∆*stpA*) more than doubled the expression of the operon (see columns 3 and 4 of Figure 3) in the cells cultured in the presence of salicin. It is possible that H-NS and StpA bind to the same site(s) and exert their repressive effects by similar or overlapping mechanisms. The effects of StpA on *bgl* operon expression documented here had not been examined in previous publications.

### 2.4. IS Insertions Promote Both the bgl Promoter and Operon Expression

**Figure 4:**
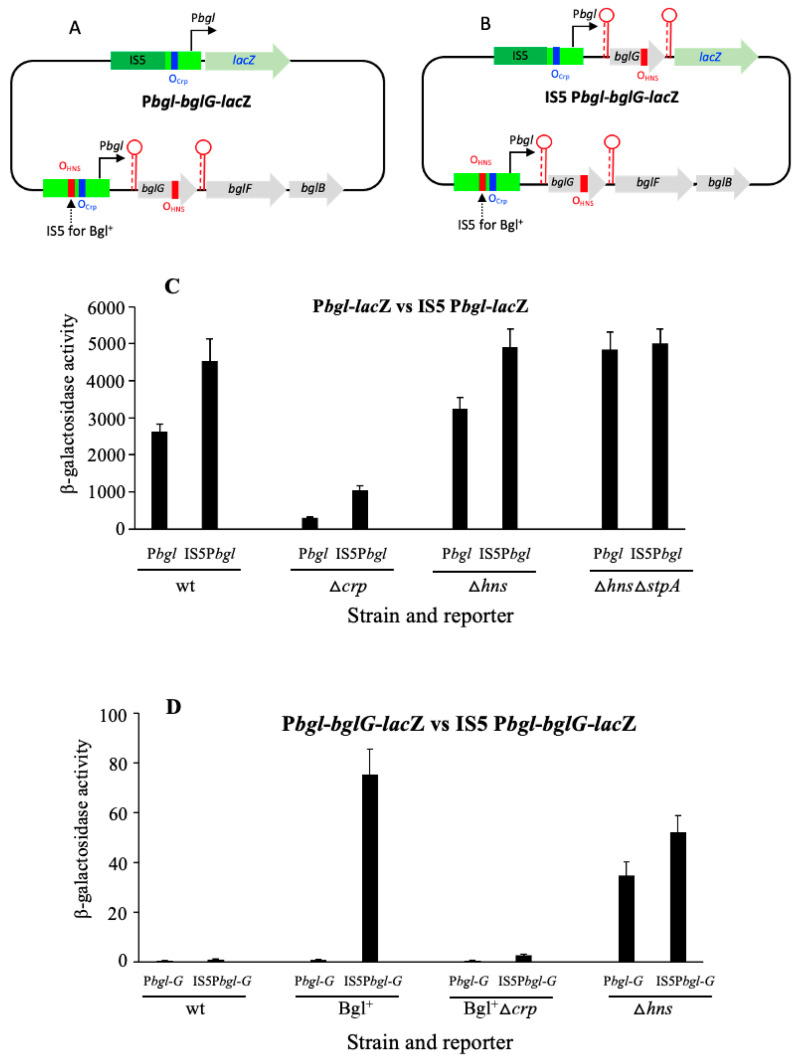
IS5 insertion stimulates both promoter and operon activities. (**A**) Diagram showing IS5 insertion at the P*bgl* reporter (IS5P*bgl*-*lacZ*). An IS5 element in the reverse direction is located at -207.5 upstream of the *bglG* translation start site. The native *bgl* operon is unchanged. (**B**) Diagram showing IS5 insertion at the *bgl* operon reporter (IS5P*bgl*-*bglG*-*lacZ*). IS5 orientation and location are the same as in Figure 4A. In both (**A**) and (**B**), the blue bars represent the Crp binding sites (O_Crp_) while the red bars represent the proposed H-NS binding sites (O_HNS_). (**C**) Effects of IS5 insertion on P*bgl* activities. (**D**) Effects of IS5 insertion on *bgl* operon activities. In both (**C**) and (**D**), test strains were cultured in M63 with glycerol and salicin as carbon sources. Sample collections and β-galactosidase assays were carried out as in Figure 1.

Figure 4A,B show a new P*bgl* reporter and a new operon reporter, respectively. They are similar to the two previously described reporters used for Figure 1, Figure 2 and Figure 3, except that an IS5 insertion was present upstream of these reporter constructs (see Section 4). For the strain Bgl^+^, the same IS5 insertion as that for the new reporter construct was present upstream of the native *bgl* operon, while for all the other test strains there was no change to the native operon.

In Figure 4C, it is evident that the presence of IS5 enhanced the reporter gene expression <2-fold in the wild type background (see the first two columns). The absence of *crp* decreased gene expression as expected, but LacZ activity was enhanced to a greater degree (about 4x) due to the IS5 insertion (see columns 3 and 4 from the left). These results suggest that with no Crp, H-NS binding is more repressive to P*bgl* since both proteins bind upstream of P*bgl*, and their binding sites may partially overlap. On the other hand, in the absence of H-NS, the IS5 insertion only moderately increased operon expression (see columns 5 and 6). However, when both *hns* and *stpA* were simultaneously deleted, there was almost no effect of IS5 on the reporter gene expression. These results suggest that with no H-NS, StpA exerts some inhibitory effects on P*bgl*.

Figure 4D shows a similar series of experiments except that the *bgl* operon was examined instead of just the promoter. In the wild type cells, the expression of *lacZ* was hardly detected, regardless of the presence or absence of the inserted IS5. This is not surprising since the native *bgl* operon remains silent and the anti-terminator protein, BglG, remains inactivated, even in the presence of salicin, the operon inducer. However, in the Bgl^+^ cells, the IS5 insertion dramatically elevated β-galactosidase activity >100 fold (see columns 3 and 4 of Figure 4D), confirming that the IS element activated the operon and rendered it highly inducible. This activity was abolished by deletion of the *crp* gene (columns 5 and 6), indicating that Crp is still essential for operon expression in the presence of salicin, even when the operon is activated by IS insertion. In other words, there are three requirements for full *bgl* operon expression: IS insertion (activating the operon), Crp (activating P*bgl*), and the operon inducer (activating BglG). Furthermore, the loss of H-NS facilitated expression with a moderate increase upon the rate of insertion of IS5 in front of the operon reporter gene construct (the last two columns of Figure 4D). This increase in activity is presumably due to StpA, which appears to exert a mild repressive effect when the *hns* gene is not present.

### 2.5. Fis Represses the Activated bgl Operon, Possibly by Interacting with Crp

**Figure 5:**
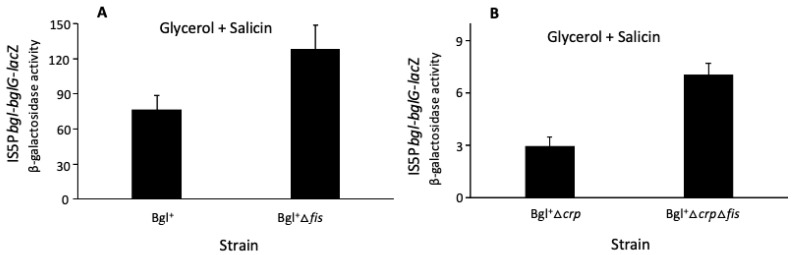
Fis represses *bgl* operon expression when it is activated by IS insertion. (**A**) Fis repression of *bgl* operon expression after activation by IS insertion. (**B**) Antagonism between Fis and Crp in regulating the *bgl* operon. In both (**A**) and (**B**), IS5 insertion is present both in the native operon and the operon *lacZ* reporter. Cells were cultured in M63 with glycerol and salicin as carbon sources. Sample collection and β-galactosidase assays were carried out as in Figure 1.

Figure 5 reveals a previously unrecognized phenomenon, namely, that Fis has a moderate repressive effect on expression of the *bgl* operon. Thus, in Figure 5A, in a Bgl^+^ background, when IS5 activates the native *bgl* operon, the deletion of *fis* gives rise to an approximately 50% increase in LacZ activity, indicating that when the *bgl* operon is activated by IS insertion, Fis is inhibitory to the operon. In the same Bgl^+^ strain, in which the *crp* gene was deleted, there is over a 2-fold increase in *bgl* operon expression when *fis* is deleted (Figure 5B), even though the total activity has decreased 20-fold compared to when Crp was present (compare Figure 5A,B). These results show that Fis exerts a repressive effect on the *bgl* operon, and the repression is even stronger when Crp is absent. Thus, while H-NS is the major repressor of the *bgl* operon, Fis and StpA are minor repressors of this operon. The nature of the degree of increase observed when Crp is absent compared with when it is present (again compare Figure 5A,B) suggests (tentatively) that Crp and Fis may exert partially, but not fully, antagonist effects.

### 2.6. DNA Looping Mediated by H-NS May Be Essential for Full bgl Operon Silencing

**Figure 6:**
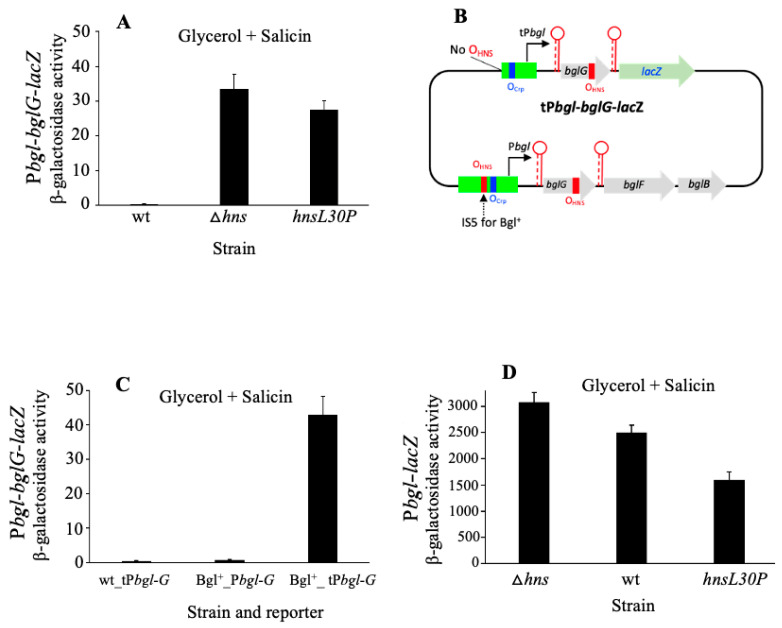
Possible requirement of DNA looping for *bgl* operon silencing. (**A**) Effect of H-NSL30P on *bgl* operon expression. H-NSL30P is an H-NS derivative that carries a proline residue instead of leucine at residue 30 in the protein. This derivative is thought to maintain its DNA-binding capability but is deficient in oligomerization [64,65], thereby failing to bridge two or more DNA loci together. Using the operon reporter, P*bgl*-*bglG*-*lacZ*, the effect of this mutant H-NS on *bgl* operon expression was determined by comparing the wild type H-NS and the absence of H-NS. (**B**) Diagram of a truncated *bgl* operon reporter (tP*bgl*-*bglG*-*lacZ*). It is the same as the regular operon reporter P*bgl*-*bglG*-*lacZ* except that the regulatory region upstream of the Crp operator (believed to carry an H-NS binding site) in P*bgl* has been removed. The blue bars represent the Crp binding sites (O_Crp_) while the red bars represent the proposed H-NS binding sites (O_HNS_). (**C**) The *bgl* operon activity using a reporter lacking the proposed H-NS binding site in the upstream regulatory region. (**D**) Effect of H-NSL30P on P*bgl*. In (**A,C**,**D**), test strains were cultured in M63 with glycerol and salicin as carbon sources at 37 °C. Sample collection and β-galactosidase assays were carried out as in Figure 1.

The results presented in Figure 6A lead to preliminary mechanistic suggestions regarding the mode of action of H-NS as a primary repressor of *bgl* operon transcription. In a wild type genetic background (with wild type H-NS), there is essentially no detectable operon expression. The deletion of the *hns* gene results in an increase in operon expression by more than 200-fold. When a mutant of H-NS is used with a single amino acid substitution at position 30 [i.e., changing a leucine to a proline (L30P)], thereby losing its self-oligomerization property and its DNA-looping activity [64,65], 15% of the repressive activity, observed in the absence of H-NS, is retained. The slightly lower operon expression seen for *hns*L30P than for ∆*hns* is most likely due to binding of the mutant H-NS to both P*bgl* and *bglG*. These results suggest that oligomerization of H-NS is important for H-NS-mediated repression. We speculate that this is because DNA looping between the two binding sites is required for the strong repressive activity of H-NS (see Section 3).

Figure 6B shows the diagram of a truncated *bgl* operon reporter (tP*bgl*-*bglG*-*lacZ*) that is essentially the same as the regular operon reporter, except that the region upstream of the Crp binding site has been removed. Conceivably, H-NS would not be able to bind to tP*bgl*. Figure 6C shows that wild type *E. coli* cells, with a wild type H-NS protein, blocks the transcription of the native *bgl* operon, and no activity was detected for the truncated operon reporter due to the presence of both terminators (column 1). Similarly, in Bgl^+^ cells, IS5 insertion activated the native *bgl* operon, but the operon reporter remained almost silent because IS5 was not present in this construct (column 2). However, using the equivalent Bgl^+^ cells carrying the truncated operon reporter (tP*bgl*-*bglG*-*lacZ*), a 60-fold increase in operon expression was seen (compare columns 2 and 3 of Figure 6C), suggesting that H-NS almost lost its repressive effect on the operon without binding, or with deficient binding, to the P*bgl* region. Thus, we believe that the binding of H-NS to BOTH of its binding sites, in the promoter region and the *bglG* gene of the reporter construct, are required for effective repression. These observations suggest that H-NS exerts its repressive effect by looping the DNA between its two binding sites (see Section 3).

To determine if the H-NS derivative, H-NSL30P, still maintained its DNA-binding capacity (despite the loss of its oligomerization property), we measured the promoter activities in cells expressing either *hns* or *hns*L30P. As seen from Figure 6D, the deletion of the *hns* gene yields the highest transcriptional activity (column 1), the restoration of H-NS function gives rise to about a 20% loss of LacZ activity (column 2), and the use of the H-NSL30P mutant protein results in the retention of the repressive activity, although it is decreased compared to the situation in which the wild type H-NS was present. These results demonstrated that both H-NS and H-NSL30P are capable of binding to the *bgl* promoter DNA, thereby repressing its activity. The decrease in transcriptional activity noted for the strain *hns*L30P when comparing the activities reported in column 2 compared with column 3 (Figure 6D) can likely be attributed to the increased binding affinity to the H-NS binding site in P*bgl* by H-NSL30P. Taken together, the significant loss of operon repression in the *hns*L30P mutant (Figure 6A) is not due to a decrease in DNA-binding activity (the mutant H-NS seems to have a greater affinity for the P*bgl* region). Instead, it is probably attributed to the failure to form a DNA loop. It seems likely that the DNA binding of H-NS gives rise to appreciable repression, but full repression is dependent on the DNA looping between the two binding sites, in the upstream promoter region and the downstream site in the *bglG* gene.

## 3. Discussion

As noted in the introductory section, the expression of the *bgl* operon has been subject to investigation by many different groups of researchers, suggesting that its regulation involves several operon-specific and global DNA-binding proteins in *E. coli* and other enteric bacteria. These studies have led to predictions as to many potential DNA-binding proteins that influence the rates of *bgl* operon transcription. However, several of these studies have reported the effects of gene overexpression on *bgl* operon activity, and consequently, some of these studies may not have physiological relevance in wild type *E. coli* cells. Thus, we have constructed reporter gene (*lacZ*) transcriptional fusions to (1) the promoter of the β-glucoside utilization operon, P*bgl*, to identify the factors that influence promoter strength, and (2) the entire regulatory region of the *bgl* operon, P*bgl*-*bglG*, including the two transcriptional terminator/anti-terminator structures flanking the *bglG* gene, in order to ascertain which factor(s) play roles in operon regulation, independently of the promoter.

The reporter gene constructs described above have been used to examine the effects of eight transcription factors (Crp, BglG, BglJ, RcsB, LeuO, H-NS, StpA, and Fis) by comparing the wild type levels of these factors under identical conditions except that each of the encoding genes had been deleted. Thus, under normal physiological conditions, we were able to gain relevant information about the involvement of the different DNA-binding proteins in the expression of P*bgl* and P*bgl*-*bglG*. The effects of the inducer, salicin, and of transposon IS5 insertion upstream of the promoter were also determined, and in several instances, we have combined the occurrence in a single strain of more than one of these factors on *bgl* transcription. For this purpose, the single *bgl*-*lacZ* constructs were expressed at one location on the chromosome while a native *bgl* operon was expressed at a distinct chromosomal location, both with and without the IS5 insertion, and with and without the *bgl* operon inducer, salicin.

The results of our studies can be summarized with the following primary conclusions. (1) The cAMP-Crp complex is the primary activator of both P*bgl* and *bgl* operon expression. (2) H-NS is a strong dominant repressor of the operon, although it is only a weak repressor of P*bgl*, irrespective of whether an inducer (salicin) is absent or present during growth. (3) The preliminary evidence suggests that H-NS exerts its repressive effect by binding to two sites and looping the DNA between these two sites. (4) StpA is a weak repressor of the *bgl* operon, but only in the absence of H-NS, suggesting that it exerts its effect independently of H-NS, contrary to a previous report [28]. (5) Fis also has a weak repressive effect on the *bgl* operon, but more so in the absence of Crp than in its presence, suggesting that there could be competition for DNA binding by these two proteins. (6) Salicin has no effect on P*bgl* activity but causes a 30-fold induction of *bgl* operon expression, probably by counteracting transcriptional termination at the two terminators flanking the *bglG* gene. (7) While P*bgl* is a strong promoter, strong transcriptional repression of the *bgl* operon occurs even under inducing conditions. (8) The inductive effect of salicin depends on the activity of the phosphoenolpyruvate-dependent BglF transporter/kinase. (9) The upstream IS5 insertion only has a moderate effect on P*bgl*, but it causes a much greater activation of *bgl* operon expression by preventing the repressive effects of H-NS and StpA. (10) While several other transcription factors have been reported to influence *bgl* operon transcription when overexpressed, they have little or no effect when present at wild type levels. These results indicate the important transcriptional regulatory mechanisms operative on the *E. coli bgl* operon while confirming or refuting several previously published suggestions and conclusions.

In this paper, we present preliminary evidence that the mechanism of H-NS repression of *bgl* transcription involves the binding of this protein to two sites in the *bgl* operon, one upstream of the promoter, and one within the *bglG* gene. However, binding to these two sites can be followed by H-NS oligomerization, possibly with the formation of a DNA loop. While DNA binding to either one or the other of its two binding sites alone can give rise to mild repression, it seems that binding alone is insufficient to cause the strong *bgl* operon repression that is caused by H-NS. Instead, our preliminary results, presented herein, suggest that the associative properties of wild type H-NS, lacking in the mutant form of the protein (H-NSL30P), are required for the strong repression that is responsible for silencing the expression of the *bgl* operon in the absence of an IS insertional event. This possibility will be the subject of a future publication, which is a work currently in progress (Lam et al., manuscript in preparation). Thus, it seems that the “on/off switch” that results from IS insertion/excision in the promoter upstream region of the *bgl* operon is largely due to the repressive effect of H-NS, and possibly, to a lesser extent, or under different conditions, due to StpA and/or Fis.

Using a *bgl*-activated strain, we showed that the deletion of *fis* caused a moderate increase in operon transcription, revealing Fis’s role as a weak repressor of the activated operon. Two Fis binding sites have been identified to overlap with the Crp binding site in P*bgl*, and in vitro assays showed that these two proteins (one repressor and one activator) compete with each other to bind to the same DNA region within P*bgl* [55]. This antagonistic relationship between Crp and Fis is supported by our in vivo assays, which demonstrated that when *crp* is deleted, Fis exhibits a greater repressive effect due to stronger binding. On the other hand, our data show that Fis has almost no effect on the *bgl* promoter. This is probably because P*bgl* is already a strong promoter, and Crp successfully outcompetes Fis to activate it. In addition, Fis and Crp exert negligible effects on the non-activated *bgl* operon in wild type cells. This is probably because with H-NS-mediated DNA looping, these DNA-binding proteins are incapable of accessing their binding sites on P*bgl*, which is embedded within the loop.

Another surprising observation concerns the *bgl* operon repression by StpA in the absence of H-NS. StpA can form heterodimers with a C-terminally truncated H-NS (still able to dimerize/ oligomerize but unable to bind DNA) to bind to the same DNA as for H-NS homodimers [51,66], suggesting that StpA homodimers alone may bind to the same DNA (that is, the *bgl* promoter and the *bglG* gene) especially when it is produced at high levels. Wolf et al. show that StpA has no appreciable inhibitory effects on upstream (that is, P*bgl*) or downstream (that is, *bglG*) silencing in the wild type or the *hns* deletion mutant [28]. In the wild type strain, the StpA level is minimal due to the strong repression by H-HS and the self-autorepression [67,68]. Therefore, it is not surprising that the deletion of *stpA* has little effect on P*bgl* and the operon expression (this study and [28]) since H-NS alone is already sufficient to silence the operon. To show that StpA does not repress the downstream site within *bglG*, Wolf et al. [28] used a reporter in which a strong constitutive promoter stimulates *bglG* and *lacZ* (only carrying the downstream regulatory element). When driven by a strong promoter, the downstream *bglG* repression by H-NS has been reported to be lost [43] as RNA polymerase can transcribe through the site bound with H-NS. A similar mechanism may explain why StpA does not exert an inhibitory effect at the *bglG* site, that is, strong transcription may help RNA polymerase pass through the site bound by StpA. In this study, we used our operon reporter, the native P*bgl* driving *bglG* and *lacZ*, which carries both upstream- and downstream-regulatory elements. The repression by H-NS via these two elements has been reported to be synergistic [43]. In the absence of H-NS, it is conceivable that high levels of StpA proteins can form enough homodimers [69], resulting in an increased (synergistic) repression to the *bgl* operon transcription, probably by binding to P*bgl* and *bglG*. Further experiments will be needed to show the direct binding of StpA homodimers to these sites within the *bgl* operon.

It is also interesting to note that even in the presence of β-glucosides in the medium during the stationary growth phase, RpoS does not appear to have an appreciable effect on *bgl* operon transcription. RpoS is required for the silencing of the *bgl* operon mediated by an H-NS mutant lacking the DNA-binding domain [57]. It has been further shown to directly repress *bgl* operon expression [56]. However, as a sigma factor, the activity of RpoS is positively affected by Crl [70], an RNA polymerase assembly factor [59]. In the absence of Crl, RpoS does not contribute to the silencing of the *bgl* operon. However, the *crl* gene is deleted from some commonly used lab *E. coli* strains including our parental strain, BW25113, which explains why RpoS did not repress *bgl* operon expression in our study.

Further studies will be required to establish the detailed repressive mechanism of this unusual operon, as well as that responsible for the very interesting process by which IS elements activate it. This class of mutations grants wild type *E. coli* cells the capacity to switch the expression of the operon using insertion sequence (IS) elements as triggers, thereby enabling the protection of the cell from toxic β-glucosides while benefiting from the presence of nutritious β-glucosides [71]. It seems likely that this is another example in which small bacterial transposons have been used to allow directed mutation to occur only under appropriate environmental stress conditions, as discussed previously [23,72,73,74,75,76,77,78,79,80].

## 4. Materials and Methods

### 4.1. E. coli Strains and Growth Media

Except for DH5**α** *pir*, used for cloning purposes, and some CGSC-deletion mutants from the *E. coli* Stock Center, all other strains used in this study were derived from K12 strain BW25113 [81], and they are described in Supplementary Table S1. Bacterial strains were routinely cultured in LB media at 30 °C or 37 °C. For β-galactosidase assays, test strains were grown in M63 minimal media with either 0.5% (*w*/*v*) glycerol, 0.5% (*w*/*v*) salicin, or both at 0.5% as carbon sources [82]. M63 salt solution contains 2 g (NH_4_)_2_SO_4_, 13.6 g KH_2_PO_4_, and 0.5 mg FeSO_4_·7H_2_O; the solution was then brought to pH 7.5 using KOH. It was supplemented with 10^−4^ % thiamine, 0.05% casamino acids, and 1.7 mM MgSO_4_. This minimal medium was used to prepare precultures and cultures prior to β-galactosidase assay. When necessary, ampicillin, kanamycin, and tetracycline were added to the media at 100 µg/mL, 25 µg/mL, and 12 µg/mL, respectively.

### 4.2. Construction of Deletion Mutants

CGSC strains JW3701-2, JW5955-1, JW2205-2, JW0075-2, JW1225, JW2644-3, and JW3229-1 (*E. coli* Genetic Stock Center, Yale Univ.) carry the deletion mutations of *bglG*, *bglJ*, *rcsB*, *leuO*, *hns*, *stpA*, and *fis*, respectively. For each of these mutants, a kanamycin resistance (km^r^) gene was substituted for the target gene. These mutations were individually transferred to strain BW25113 (wild type; [81]) by P1 transduction, and the km^r^ gene was subsequently flipped out by pCP20 [81], yielding the deletion mutant strains ∆*bglG*, ∆*bglJ*, ∆*rcsB*, ∆*leuO*, ∆*hns*, ∆*stpA* and ∆*fis*, respectively (Supplementary Table S1). The *bglJ* mutation was transferred into strain ∆*rcsB*, yielding the ∆*bglJ*∆*rcsB* double mutant. The *stpA* mutation was transferred into strain ∆*hns*, yielding the ∆*hns*∆*stpA* double mutant.

### 4.3. Construction of the bgl Promoter Transcriptional Reporter Pbgl-lacZ

To create the *bglGFB* promoter-*lacZ* transcriptional fusion used to measure the promoter activities, the promoter region (−205 to + 54 relative to the transcriptional start site, + 1) without the first terminator upstream of *bglG*, was amplified using oligos Pbgl-Xho-F and Pbgl-Bam-R (Supplementary Table S2), digested with XhoI and BamHI, and then cloned into the same XhoI/BamHI sites of the integration vector, pKDT [83], yielding pKDT-P*bgl*. The region carrying the *km*^r^, *rrnB*T and P*bgl* (*km*^r^:*rrnB*T:P*bgl*) was PCR-amplified using oligos bgl-Z-P1 and Pbgl-Z-P2 (Supplementary Table S2) and then integrated into the chromosomal default strain EQ42 [83] to replace the *lacI* gene and the *lacZ* promoter. The resultant strain carried the *km*^r^:*rrnB*T:P*bgl* cassette followed by *lacZ*’s ribosomal binding site (RBS) and the *lacZ* structural gene within the *lac* locus. After being confirmed by PCR and sequencing, the promoter reporter, P*bgl* driving *lacZ* expression (that is, P*bgl*-*lacZ*) was transferred into BW25113 and various genetic backgrounds by P1 transduction. This yielded the *bgl* promoter reporter strains BW_P*bgl*-Z, ∆*bglG*_P*bgl*-Z, ∆*bglJ*_P*bgl*-Z, ∆*rcsB*_P*bgl*-Z, ∆*leuO*_P*bgl*-Z, ∆*hns*_P*bgl*-Z, ∆*stpA*_P*bgl*-Z, ∆*fis*_P*bgl*-Z, ∆*bglJ*∆*rcsB*_P*bgl*-Z, and ∆*hns*∆*stpA*_P*bgl*-Z, respectively. The P*bgl*-Z reporter was transferred into a *crp* deletion mutant [73], yielding the strain ∆*crp*_P*bgl*-Z. In addition, the same reporter was transferred into one previously isolated Bgl^+^ mutant (carrying a reverse-oriented IS5 element at -207.5, located upstream of the *bglG* translation start site), yielding the strain Bgl^+^_P*bgl*-Z (Supplementary Table S1). To determine how an IS5 insertion affects P*bgl* activities, the regulatory region carrying both IS5 (the same IS5 as for Bgl^+^) and P*bgl* was PCR-amplified using the same oligos as for P*bgl* amplification from the genomic DNA of Bgl^+^ cells. The resultant product, IS5P*bgl*, was cloned into pKDT, yielding pKDT-IS5P*bgl*. The IS5P*bgl* cassette was chromosomally integrated within the *lac* locus as described above for the P*bgl* cassette. This promoter reporter was transferred into BW25113, ∆*crp*, ∆*hns*, and ∆*hns*∆*stpA*, yielding the strains BW_IS5P*bgl*-Z, ∆*crp*_IS5P*bgl*-Z, ∆*hns*_IS5P*bgl*-Z, and ∆*hns*∆*stpA*_IS5P*bgl*-Z, respectively.

### 4.4. Construction of the bgl Operon Transcriptional Reporter Strains

Recently, we reported the construction of a *bgl* operon transcriptional reporter P*bgl*-*bglG*-*lacZ* (referred to as G-Z) [23]. Located within the *lac* locus, this operon reporter construct carries the *bglGFB* promoter and the first gene, *bglG*, including the 2nd terminator downstream of the *bglG* translational stop codon (the 205th nucleotide to the 1127th nucleotide relative to the transcriptional start site) followed by a stop codon, *lacZ*’s RBS, and the *lacZ* structural gene. In addition to three operon reporter strains (BW_Z, P*tet*-G_Z, and Iq-G_Z), this operon reporter was transferred by P1 transduction to other genetic backgrounds. This yielded Bgl^+^_G-Z, ∆*crp*_G-Z, ∆*bglG*_G-Z, ∆*bglJ*_G-Z, ∆*rcsB*_G-Z, ∆*bglJ*∆*rcsB*_G-Z, ∆*leuO*_G-Z, ∆*hns*_G-Z, ∆*stpA*_G-Z, ∆*fis*_G-Z, ∆*hns*∆*stpA*_G-Z. To examine the effect of the IS insertion on expression of the *bgl* operon, a new *bgl* operon transcriptional reporter, IS5P*bgl*-*bglG*-*lacZ* (referred as IS5G-Z), was constructed, in which a *reverse*-oriented IS5 element was inserted upstream of the original operon reporter P*bgl*-*bglG*-*lacZ* (the same IS5 as for Bgl^+^). To achieve this, the regulatory region containing IS5 and P*bgl*-*bglG* was amplified from the Bgl^+^ cells (containing the reverse-oriented IS5 at the same location as for the operon reporter) using the same oligos as for P*bgl*-*bglG*. The resultant IS5P*bgl*-*bglG* product was cloned into pKDT, yielding pKDT_IS5P*bgl*-*bglG*. The IS5P*bgl*-*bglG* cassette was chromosomally integrated within the *lac* locus as recently reported in [23]. This operon reporter was transferred to BW25113, Bgl^+^, ∆*crp*, ∆*hns*, Bgl^+^∆*fis*, Bgl^+^∆*crp*, and Bgl^+^∆*crp*∆*fis*, yielding operon reporter strains BW25113_IS5G-Z, Bgl^+^_IS5G-Z, ∆*crp*_IS5G-Z, ∆*hns*_IS5G-Z, Bgl^+^∆*fis*_IS5G-Z, Bgl^+^∆*crp*_IS5G-Z and Bgl^+^∆*crp*∆*fis*_IS5G-Z, respectively. To make a truncated operon transcriptional reporter (tP*bgl*-*bglG*-*lacZ* or referred to as tG-Z), a smaller DNA region (−93 to + 1127 relative to the transcriptional start site, + 1), supposedly not carrying the H-NS binding site on *Pbgl*, was cloned into pKDT, yielding pKDT-tP*bgl-bglG*. This DNA fragment, “*km*^r^:rrnBT:tPbgl-bglG”, was chromosomally integrated within the same *lac* locus as for P*bgl*-*bglG*-*lacZ* [23], yielding tP*bgl*-*bglG*, which drives *lacZ* expression. This truncated operon reporter was transferred into BW25113 and Bgl^+^, yielding the strains BW25113_tG-Z and Bgl^+^_tG-Z, respectively.

### 4.5. Construction of the hnsL30P Strain Using Positive/Negative Selection

H-NS is the primary silencer of the *bgl* operon. The H-NS protein usually exists in oligomeric forms, and these contribute to its biological activity [84], promoting the formation of structures such as DNA loops and bridges [40,85]. The N-terminal domain is responsible for H-NS oligomerization. The leucine residue at position 30 is essential for H-NS:H-NS binding [84]. To test the possible looping mechanism by which H-NS silences the *bgl* operon, we used a two-step recombineering protocol based on TetA-SacB positive-selection and counter-selection [86] to change the leucine codon CTG (88 to 90 relative to the first *hns* codon ATG) to a proline codon CCT in the *hns* gene. “TG” in the leucine codon was first replaced by the *tetA*-*sacB* cassette that was amplified from the chromosomal DNA of strain T-SACK [86] using chimeric oligos hns-AB-F and hns-AB-R (Supplementary Table S2). These long oligos carry the appropriate homologous arms flanking the “TG” nucleotides in the *hns* gene. The replacement of “TG” by *tetA*-*sacB* in some tetracycline (Tc)-resistant mutants was confirmed by colony PCR and sequencing. A 100-bp single strand DNA fragment, which covers the region of the *hns* gene (38 to 138 relative to the ATG) with CT replacing “TG” in the middle, was synthesized. This fragment was amplified using oligos hns-F and hns-R (Supplementary Table S2), and PCR products were electroporated into the cells of a Tc resistant mutant expressing Lambda-Red proteins encoded by pKD46 [81]. After one-hour incubation, the electroporated cells were applied onto TetA/SacB counter-selection agar (plus 6% sucrose and 24 mg fusaric acid per liter). After incubation at 42 °C for about two days, 10 colonies resistant to sucrose and fusaric acid were purified on LB agar plates and tested for both sensitivity to Tc and resistance to sucrose. Several Tc-sensitive/sucrose-resistant colonies were confirmed for the replacement of the *tetA*-*sacB* cassette with “CT” by PCR and sequencing. The resultant altered strain was named *hns*L30P, in which the 30th codon was changed from leucine to proline.

### 4.6. β-Galactosidase Assays

*E. coli* reporter strains were cultured in 4 mL of LB contained in glass test tubes (1.5 cm in diameter × 15 cm in length) with shaking at 37 °C for 8 h. An amount of 30 µL of LB cultures were used to inoculate 3 mL of M63 minimal media in smaller glass tubes (1.2 cm × 12 cm), and the tubes were shaken at 37 °C overnight. The carbon sources were 0.5% glycerol, 0.5% salicin, or both. To improve the growth of the hns-deletion mutant and its derivatives, casamino acids (CAA) were added to all minimal M63 media to 0.05%. The overnight M63 cultures (precultures) were inoculated into 5 mL of the same media in larger test tubes (1.8 cm × 15 cm) with an initial OD_600_ of 0.03. The tubes were rotated at 250 rpm and 37 °C, and cell densities (OD_600_) were measured with a Bio-Rad spectrophotometer. During the exponential growth phase, four samples were collected in the range of OD_600_ from 0.1 to 1. The samples (roughly 0.3 mL for promoter reporter strains, and 0.6 mL for operon reporter strains) were immediately frozen at −20 °C prior to β-galactosidase assays. To test RpoS effects, samples were collected in the range of OD_600_ from 1 to 4 when the cultures entered the early and late stationary phases.

To measure β-galactosidase activities in *bgl* promoter reporter strains, 0.8 mL of Z-buffer containing β-mercaptoethanol (2.7 μL/mL) and sodium dodecyl sulfate (SDS) (0.005%) was mixed with 0.2 mL of sample and 25 μL of CHCl_3_ in test tubes. Alternatively, for *bgl* operon reporter strains, 0.5 mL of Z-buffer was mixed with 0.5 mL of the sample. The tubes were vortexed twice (each time for 10 s at a constant speed) and incubated in a 37 °C water bath until equilibration. A 0.2 mL aliquot of O-nitrophenyl galactoside (ONPG) substrate (4 mg/mL) was then added to each test tube. When a yellow color developed, the reaction was stopped by adding 0.5 mL of 1 M Na_2_CO_3_ followed by vortexing. Reaction mixtures were centrifuged (15,000 rpm, 3 min), and the absorbance values of the supernatants were measured at 420 nm and 550 nm. A control tube was run in parallel using M63 salts instead of the test sample. β-galactosidase activity (Miller units) = [(OD_420_−1.75 × OD_550_)/(sample volume in mL × time in min)] × 1000 [87]. For a given test strain, the slope of OD_600_ values versus β-galactosidase activities was referred to as the promoter activity or the operon activity.

## Data Availability

Not applicable.

## Supplementary Materials

The following supporting information can be downloaded at: https://www.mdpi.com/article/10.3390/ijms231810343/s1.
