# Supplementary material for: Effects of Global and Specific DNA-Binding Proteins on Transcriptional Regulation of the E. coli bgl Operon
Source: Int J Mol Sci. 2022 Sep 7;23(18):10343. doi: 10.3390/ijms231810343 (PMC9499468; doi:10.3390/ijms231810343)
Supplement: Supplementary file 1 [file ijms-23-10343-s001.zip › ijms-1869932-supplementary.pdf]

Supplementary Data

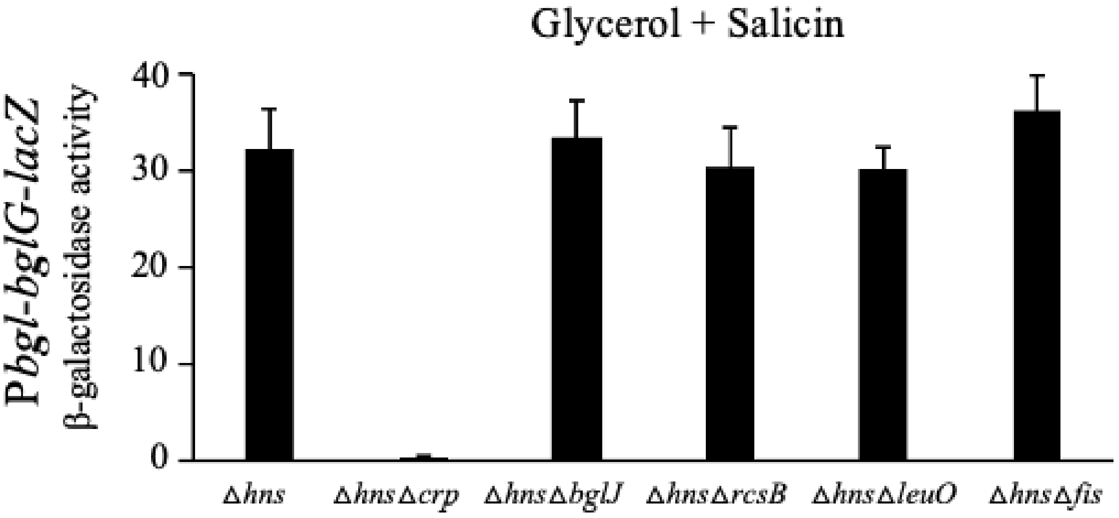

Figure S1. Effects of various transcription factors on *bgl* operon activities in the absence of H-NS. The genes encoding transcription regulators were individually deleted in a *hns* deletion background. Using the operon reporter P*bgl*-*bglG*-*lacZ*, the bgl operon activities from all the double mutants were measured. Strain cultures, sample collections and β-galactosidase assays were carried out as for Figure 1.

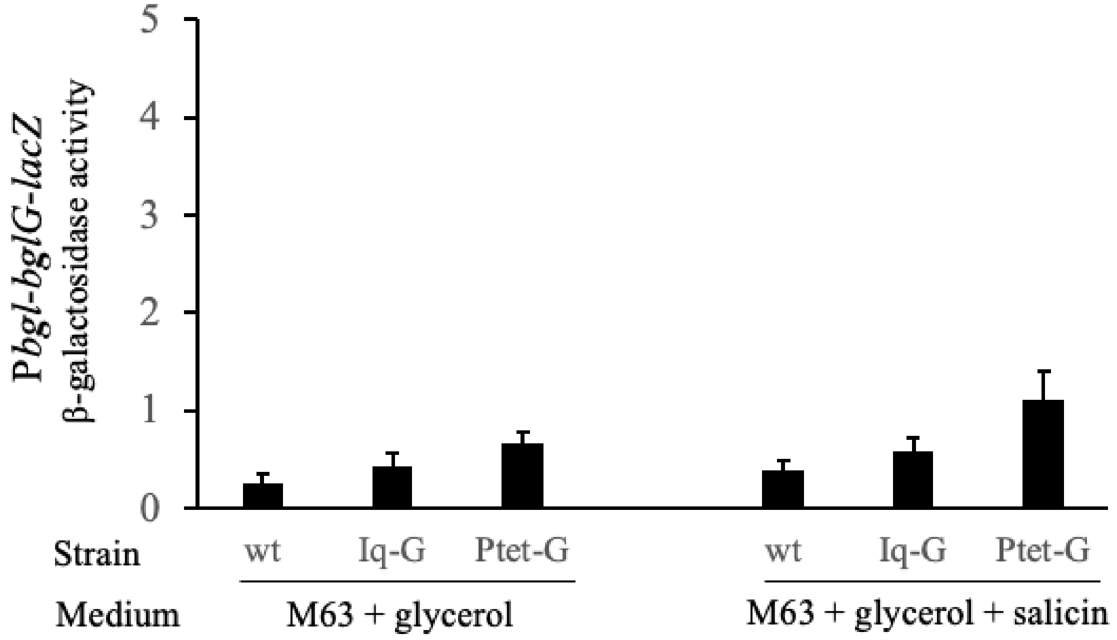

Figure S2. H-NS repression of *bgl* operon expression in cells over-expressing *bglG*. The *bglG* gene was driven by constitutive promoters *lacI*q (medium strength) and P*tet* (strong) at the *intS* locus. Using P*bgl*-*bglG*-*lacZ* as the reporter, the operon activities were measured when cells were cultured in M63 with glycerol only (left) or glycerol and salicin (right). Sample collection and β-galactosidase assays were carried out as for Figure 1.

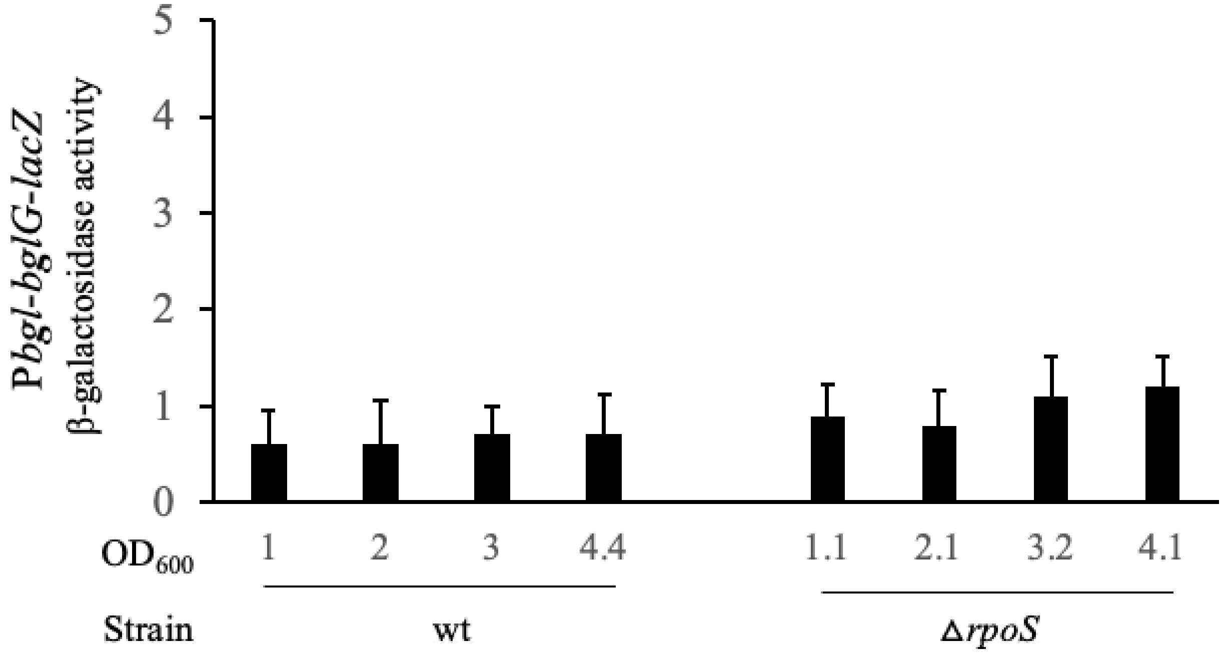

Figure S3. Effect of an *rpoS* deletion mutation on *bgl* operon expression as the cells approach and enter the stationary growth phase. Using P*bgl-bglG-lacZ* as the reporter, the operon activities were measured when wild type and Δ*rpoS* cells were cultured in M63 with glycerol and salicin at 37°C. Samples were collected when the OD_{600} reached about 1, 2, 3 and 4, respectively. β-galactosidase assays were carried out as for Figure 1.

Supplementary Table S1. Strains and plasmids used in this study

Strains or plasmids	Genotype or description	Reference
**Strains**		
BW25113	Wild type, *lacI*^{q} *rrnB*$_{T14}$ Δ*lacZ*$_{WJ}$ Δ*hsdR*514 Δ*araBAD*$_{AH33}$ Δ*rhaBAD*$_{LD78}$	[1]
Bgl^{+}	IS5 in a reverse orientation at -207.5 upstream of *bglG* translation start site in BW25113	This study
Δ*crp*	Deletion of *crp* in BW25113	[2]
Δ*bglG*	Deletion of *bglG* in BW25113	This study
Δ*bglJ*	Deletion of *bglJ* in BW25113	This study
Δ*rcsB*	Deletion of *rcsB* in BW25113	This study
Δ*bglJ*Δ*rcsB*	Deletion of *bglJ* and *rcsB* in BW25113	This study
Δ*leuO*	Deletion of *leuO* in BW25113	This study
Δ*hns*	Deletion of *hns* in BW25113	This study
Δ*stpA*	Deletion of *stpA* in BW25113	This study
Δ*hns*Δ*stpA*	Deletion of *hns* and *stpA* in BW25113	This study
Δ*fis*	Deletion of *fis* in BW25113	This study
Iq-G	*lacIq* driving *bglG* at the *intS* locus in BW25113	[3]
Ptet-G	P*tet* driving *bglG* at the *intS* locus in BW25113	[3]
BW_P*bgl*-Z	P*bgl* driving *lacZ* at the *lac* locus in BW25113	This study
Bgl^{+}_P*bgl*-Z	P*bgl* driving *lacZ* at the *lac* locus in Bgl^{+}	This study
Δ*crp*_P*bgl*-Z	P*bgl* driving *lacZ* at the *lac* locus in Δ*crp*	This study
Δ*bglG* _P*bgl*-Z	P*bgl* driving *lacZ* at the *lac* locus in Δ*bglG*	This study
Δ*bglJ*_P*bgl*-Z	P*bgl* driving *lacZ* at the *lac* locus in Δ*bglJ*	This study
Δ*rcsB*_P*bgl*-Z	P*bgl* driving *lacZ* at the *lac* locus in Δ*rcsB*	This study
Δ*bglJ*Δ*rcsB*_P*bgl*-Z	P*bgl* driving *lacZ* at the *lac* locus in Δ*bglJ*Δ*rcsB*	This study
Δ*leuO*_P*bgl*-Z	P*bgl* driving *lacZ* at the *lac* locus in Δ*leuO*	This study
Δ*hns*_P*bgl*-Z	P*bgl* driving *lacZ* at the *lac* locus in Δ*hns*	This study
Δ*stpA*_P*bgl*-Z	P*bgl* driving *lacZ* at the *lac* locus in Δ*stpA*	This study
Δ*fis*_P*bgl*-Z	P*bgl* driving *lacZ* at the *lac* locus in Δ*fis*	This study
BW25113_G-Z	P*bgl-bglG* driving *lacZ* at the *lac* locus in BW25113	[3]
Bgl^{+}_G-Z	P*bgl-bglG* driving *lacZ* at the *lac* locus in Bgl^{+}	This study
Δ*crp*_G-Z	P*bgl-bglG* driving *lacZ* at the *lac* locus in Δ*crp*	This study
Δ*bglG*_G-Z	P*bgl-bglG* driving *lacZ* at the *lac* locus in Δ*bglG*	This study
Δ*bglJ*_**G-**Z	P*bgl-bglG* driving *lacZ* at the *lac* locus in Δ*bglJ*	This study
Δ*rcsB*_G-Z	P*bgl-bglG* driving *lacZ* at the *lac* locus in Δ*rcsB*	This study
Δ*bglJ*Δ*rcsB*_G-Z	P*bgl-bglG* driving *lacZ* at the *lac* locus in Δ*bglJ*Δ*rcsB*	This study
Δ*leuO*_G-Z	P*bgl-bglG* driving *lacZ* at the *lac* locus in Δ*leuO*	This study
Δ*hns*_G-Z	P*bgl-bglG* driving *lacZ* at the *lac* locus in Δ*hns*	This study
Δ*stpA*_G-Z	P*bgl-bglG* driving *lacZ* at the *lac* locus in Δ*stpA*	This study
Δ*fis*_G-Z	P*bgl-bglG* driving *lacZ* at the *lac* locus in Δ*fis*	This study
Δ*hns*Δ*stpA*_G-Z	P*bgl-bglG* driving *lacZ* at the *lac* locus in Δ*hns*Δ*stpA*	This study
Ptet-G_G-Z	P*bgl-bglG* driving *lacZ* at the *lac* locus in Ptet-G	[3]
Iq-G_G-Z	P*bgl-bglG* driving *lacZ* at the *lac* locus in Iq-G	[3]
BW_IS5P*bgl*-Z	IS5P*bgl* driving *lacZ* at the *lac* locus in BW25113	This study
Δ*crp*_IS5P*bg*l-Z	IS5P*bgl* driving *lacZ* at the *lac* locus in Δ*crp*	This study
Δ*hns*_IS5P*bgl*-Z	IS5P*bgl* driving *lacZ* at the *lac* locus in Δ*hns*	This study
Δ*hns*Δ*stpA*_IS5P*bgl*-Z	IS5P*bgl* driving *lacZ* at the *lac* locus in Δ*hns*Δ*stpA*	This study
BW25113_IS5G-Z	IS5P*bgl-bglG* driving *lacZ* at the *lac* locus in BW25113	This study
Bgl^{+}_IS5G-Z	IS5P*bgl-bglG* driving *lacZ* at the *lac* locus in Bgl^{+}	This study
Δ*crp*_IS5G-Z	IS5P*bgl-bglG* driving *lacZ* at the *lac* locus in Δ*crp*	This study
Δ*hns*_IS5G-Z	IS5P*bgl-bglG* driving *lacZ* at the *lac* locus in Δ*hns*	This study

Bgl$^+$Δ*fis*	Deletion of *fis* in Bgl$^+$	This study
Bgl$^+$Δ*crp*	Deletion of *crp* in Bgl$^+$	This study
Bgl$^+$Δ*crp*Δ*fis*	Deletion of *crp* and *fis* in Bgl$^+$	This study
Bgl$^+$Δ*fis*_IS5G-Z	IS5P*bgl-bglG* driving *lacZ* at the *lac* locus in Bgl$^+$Δ*fis*	This study
Bgl$^+$Δ*crp*_IS5G-Z	IS5P*bgl-bglG* driving *lacZ* at the *lac* locus in Bgl$^+$Δ*crp*	This study
Bgl$^+$Δ*crp*Δ*fis*_IS5G-Z	IS5P*bgl-bglG* driving *lacZ* at the *lac* locus in Bgl$^+$Δ*crp*Δ*fis*	This study
*hns*L30P	Mutation of leucine to proline at 30th codon in the *hns* gene	This study
*hns*L30P_P*bgl*-Z	P*bgl* driving *lacZ* at the *lac* locus in *hns*L30P	This study
*hns*L30P_G-Z	P*bgl-bglG* driving *lacZ* at the *lac* locus in *hns*L30P	This study
BW25113_tG-Z	A truncated P*bgl-bglG* driving *lacZ* at the *lac* locus in BW25113	This study
Bgl$^+$_tG-Z	A truncated P*bgl-bglG* driving *lacZ* at the *lac* locus in Bgl$^+$	This study
**Plasmids**		
pKDT	A *rrnB* terminator (*rrnB*T) in pKD13	[4]
pKDT_P*bgl*	P*bgl* cloned downstream of the *rrnB*T in pKDT	This study
pKDT_IS5P*bgl-bglG*	IS5P*bgl-bglG* cloned downstream of the *rrnB*T in pKDT	This study
pKDT-tP*bgl-bglG*	tP*bgl-bglG* cloned downstream of the *rrnB*T in pKDT	This study

References

1. Datsenko, K. A.; Wanner, B. L., One-step inactivation of chromosomal genes in *Escherichia col*i K-12 using PCR products. *Proc Natl Acad Sci U S A* **2000,** 97, (12), 6640-5. DOI: 10.1073/pnas.120163297. PMID: 10829079.
2. Zhang, Z.; Saier, M. H., Jr., A mechanism of transposon-mediated directed mutation. *Mol Microbiol* **2009,** 74, (1), 29-43. DOI: 10.1111/j.1365-2958.2009.06831.x. PMID: 19682247.
3. Zhang, Z.; Zhou, K.; Tran, D.; Saier, M., Insertion Sequence (IS) Element-Mediated Activating Mutations of the Cryptic Aromatic beta-Glucoside Utilization (BglGFB) Operon Are Promoted by the Anti-Terminator Protein (BglG) in *Escherichia coli*. *Int J Mol Sci* **2022,** 23, (3). DOI: 10.3390/ijms23031505. PMID: 35163427.
4. Klumpp, S.; Zhang, Z.; Hwa, T., Growth rate-dependent global effects on gene expression in bacteria. *Cell* **2009,** 139, (7), 1366-75. DOI: 10.1016/j.cell.2009.12.001. PMID: 20064380.

Supplementary Table S2. Oligonucleotides used in this study

Name	Sequence	Use
Pbgl-Xho-F	atactcgagtggcgatgagctggataaactgctg	Cloning P*bgl*, P*bgl*-*bglG* or IS5P*bgl*-*bglG* into pKDT
Pbgl-Bam-R	ttaggatcctgcagtaacaatccagtcatttattaatg	Cloning P*bgl* into pKDT
bglG-Bam-R	taggatccttagactatttttctggctaactccgtc	Cloning P*bgl*-*bglG*, IS5P*bgl*-*bglG* or tP*bgl*-*bglG* into pKDT
tPbgl-Xho-F	aatctcgagctaaaattacacaaagttaataactg	Cloning tP*bgl*-*bglG* into pKDT
Pbgl-F2	tggcgatgagctggataaactg	Verification of P*bgl* cloning
Pbgl-R2	tcagttcatgactgctcaaggcatac	Verification of P*bgl* cloning
bglG-ver-F1	agcatggcaatgtgcggaacggatc	Verification of P*bgl*-*bglG* cloning
bglG-ver-F2	ttgtggtggttattgatgatcaacag	Verification of P*bgl*-*bglG* cloning
bglG-ver-R1	taactgcgtgacacctgcaacatc	Verification of P*bgl*-*bglG* cloning
bglG-ver-R2	tcccaagcgctcctgcgctaaagag	Verification of P*bgl*-*bglG* cloning
IS5R-ver-R1	accttatccgctggaaaccatgctac	Verification of IS5P*bgl*-*bglG* cloning
IS5R-ver-R2	aaggtaagaaccttgaaacagcatc	Verification of IS5P*bgl*-*bglG* cloning
bgl-Z-P1	gcatttacgttgacaccatcgaatggcgcaaaacctttcgcggtatgtgtaggctgga gctgcttc	Amplification of P*bgl*, P*bgl*-*bglG* or IS5P*bgl*-*bglG* with *km*^{r} from pKDT_P*bgl*, pKDT_P*bgl*-*bglG* or pKDT_IS5P*bgl*-*bglG*
Pbgl-Z-P2	cgacggccagtgaatccgtaatcatggtcatagctgtttcctgtgtgaaatgcagtaa caatccagtcatttattaatg	Amplification of P*bgl* with *km*^{r} from plasmid pKDT_P*bgl*
bglG-Z2	cgacggccagtgaatccgtaatcatggtcatagctgtttcctgtgtgaaattagactat ttttctggctaactcc	Amplification of P*bgl*-*bglG*, IS5P*bgl*-*bglG* or IS5P*bgl*-*bglG* with *km*^{r} from pKDT_P*bgl*-*bglG*, pKDT_IS5P*bgl*-*bglG* or pKDT_tP*bgl*-*bglG*
hns-AB-F	ctcttcgtgcgcaggcaagagaatgtacacttgaaacgctggaagaaatgctcctaa tttttgttgacactctatc	Amplification and chromosomal integration of *tetA*:*sacB*
hns-AB-R	agccgcgctttcttcttcgcgacgttcgttaacaacaacttctaatttttcatcaaaggg aaaactgtccatatgc	Amplification and chromosomal integration of *tetA*:*sacB*
hns-100	cttcgtgcgcaggcaagagaatgtacacttgaaacgctggaagaaatg**cct**gaaaa attagaagttgttgttaacgaacgtcgcgaagaagaaagcgcgg	A partial *hns* fragment carrying the L30P mutation (bolded)
hns-F	cacttaaaattctgaacaacatccgtactcttcgtgcgcaggcaagagaatg	Amplification of hns-100
hns-R	gagtgcgctcttcaacttcagcagcagccgcgctttcttcttcgcgacgttc	Amplification of hns-100
hns-ver-R	agattattgcttgatcaggaaatcgtc	Verification of *hns*L30P on chromosome
